# HTLV-1 inserts an ectopic CTCF-binding site into the human genome

**DOI:** 10.1186/1742-4690-12-S1-P12

**Published:** 2015-08-28

**Authors:** Yorifumi Satou, Miyazato Paola, Ko Ishihara, Asami Fukuda, Kisato Nosaka, Takehisa Watanabe, Aileen Rowan, Mitsuyoshi Nakao, Charles RM Bangham

**Affiliations:** 1 Department of Immunology, Imperial College London, London W2 1PG, UK; 2 Kumamoto University, Kumamoto, 860-0811, Japan; 3 International Research Center for Medical Sciences (IRCMS), Kumamoto University, Kumamoto, 860-0811, Japan

## 

HTLV-1 genes are encoded on both strands of the provirus, such as tax in the plus and HBZ in the minus strand. The HBZ gene is constitutively expressed from the negative strand of the integrated provirus, whereas plus-strand expression, required for viral propagation to uninfected cells, is expressed only intermittently in vivo, perhaps to escape from host immune surveillance. However, it remains unknown what regulates this pattern of proviral transcription in vivo. We have found that CTCF binds to the HTLV-1 provirus. CTCF is a DNA-binding protein that plays a fundamental role in controlling higher-order chromatin structure and gene expression in vertebrates. We identified several candidate regions for CTCF binding in the HTLV-1 genome. Chromatin immunoprecipitation assays showed that CTCF bound selectively to the pX region of HTLV-1. Furthermore, electromobility shift assays revealed that CTCF bound directly to the pX DNA sequence. Consistent with the CTCF binding, there was a sharp border of histone modification patterns at the pX region, consistent with CTCF's role as a chromatin insulator. Finally, the CTCF-binding region (1bp) showed enhancer-blocking activity. The CTCF binding and epigenetic border were detectable not only in HTLV-1 cell lines and ATL cell lines but also in fresh PBMCs of ATL patients. These observations suggest that CTCF plays a central role in the regulation of HTLV-1 transcription.

#Poster award winnder - 1st place

